# Nrf2 enhances the therapeutic efficiency of adipose-derived stem cells in the treatment of neurogenic erectile dysfunction in a rat model

**DOI:** 10.1186/s12610-023-00214-x

**Published:** 2023-12-20

**Authors:** Shangbin Yang, Wancheng Shi, Qianhui Liu, Yingqiu Song, Jiafeng Fang

**Affiliations:** 1 Department of Gastrointestinal Surgery, The Third Affiliated Hospital of Sun Yat-Sen University, Tianhe Road 600, Guangzhou, 510630 China; 2 Department of Gastrointestinal Surgery, Shenshan Medical Center, Sun Yat-sen Memorial Hospital, Sun Yat-sen University, Shanwei, 516621 China

**Keywords:** Nuclear factor-E2 related factor, Erectile dysfunction, Bilateral cavernous nerve injury, Cell therapy, Nrf2, Dysfonction erectile, Lésion bilatérale du Nerf caverneux, Thérapie cellulaire

## Abstract

**Background:**

Erectile dysfunction (ED) caused by intraoperative nerve injury is a major complication of pelvic surgery. Adipose-derived stem cells (ADSCs) have presented therapeutic potential in a rat model of bilateral cavernous nerve injury (BCNI), while inadequate in vivo viability has largely limited their application. Nuclear factor-E2-related Factor (Nrf2) is a key transcription factor that regulates cellular anti-oxidative stress. In this work, we investigated the effect of Nrf2 expression regulation on the viability of ADSCs, and explore its repair potential in a BCNI rat model.

**Results:**

The survival time of tert-Butylhydroquinone (tBHQ)-ADSCs in BCNI model increased obviously. In addition, the tBHQ-ADSCs group presented better restoration of major pelvic ganglion (MPG) nerve contents and fibers, better improvement of erectile function, and less penile fibrosis than the other groups. Moreover, the expression of Nrf2 and superoxide dismutase 1 (SOD1) were higher than those of other groups.

**Conclusion:**

Nrf2 could enhance the anti-oxidative stress ability of ADSCs, so as to improve the therapeutic effect of ADSCs on BCNI rat model.

## Résumé

**Contexte:**

La dysfonction érectile (DE) causée par une lésion nerveuse peropératoire est une complication majeure de la chirurgie pelvienne. Les cellules souches dérivées du tissu adipeux (ADSC) ont constitué un potentiel thérapeutique dans un modèle de lésion bilatérale des nerfs caverneux (BCNI) chez le rat, mais une viabilité insuffisante in vivo a largement limité leur application. Nrf2 est un facteur de transcription clé qui régule le stress antioxydant cellulaire. Dans ce travail, nous avons étudié l’effet de la régulation de l’expression de Nrf2 sur la viabilité des ADSC, et exploré son potentiel de réparation dans un modèle de BCNI chez le rat.

**Résultats:**

Le temps de survie des cellules tert-butylhydroquinone (tBHQ)-ADSC dans le modèle BCNI a significativement augmenté. De plus, le groupe tBHQ-ADSC a présenté une meilleure restauration du contenu et des fibres nerveuses des ganglions pelviens majeurs, une meilleure amélioration de la fonction érectile et une moindre fibrose pénienne que les autres groupes. Par ailleurs, l’expression de Nrf2 et de la superoxyde dismutase 1 était plus élevée dans ce groupe que dans les autres groupes.

**Conclusions:**

Nrf2 pourrait améliorer la capacité de stress anti-oxydatif des cellules ADSC, améliorant ainsi l’effet thérapeutique des ADSC sur le modèle de BCNI chez le rat.

## Introduction

Pelvic autonomic nerve injury is a common complication of pelvic surgery, and nerve injury-induced erectile dysfunction (ED) has led to great impairment of postoperative quality of life. Although pelvic autonomic nerve preservation surgeries have been widely performed, nerve injury is still inevitable in some cases, resulting in a continued high incidence of ED [1]. Phosphodiesterase type 5 inhibitors (PDE5Is) are the most common used treatment for various types of ED, including aging and diabetes. However, the efficacy of PDE5Is in the treatment of neurotraumatic ED is not sufficient, as neurotraumatic ED progresses gradually due to denervation and progressive fibrosis [2].

Therefore, there is an urgent need to explore effective treatments. Mesenchymal stem cells (MSCs) have been shown to have therapeutic effects on neurotraumatic ED in rats in several studies [3–5]. After implantation, MSCs can secrete neurotrophic factors, growth factors and other bioactive substances to protect injured nerves and promote nerve regeneration [6, 7]. Thus, MSCs transplantation has become a potential competitive candidate in therapy of neurotraumatic ED.However, the survival ability of MSCs in vivo is short, which largely affects its therapeutic effect. An important reason for inadequate efficiency of MSCs in vivo is oxidative stress injury. Due to the high oxidative stress at the nerve injury site, the activity of transplanted cells is significantly inhibited [8, 9]. Nuclear factor-E2-related Factor (Nrf2) is a key transcription factor regulating the cellular antioxidant system, and can regulate antioxidant enzymes such as glutathione transferase and nicotinamide adenine dinucleotide phosphate (NAD(P)H), thereby affecting cell metabolism [10–12]. Therefore, we hypothesized that regulating Nrf2 expression could enhance the antioxidant capacity of transplanted MSCs, thereby increasing the activity of MSCs and enhancing their effects at the transplant site. In this study, we aimed to investigate the effect of Nrf2 on enhancing the viability of MSCs, so as to improve the therapeutic effect of MSCs on nerve injured-induced ED rat models.

## Methods and materials

### Ethics

This study was approved by the Hospital Animal Research Committee of Sun Yat-sen University (SYSU-IACUC-2021–000609). All experiments were performed in accordance with their approved guidelines.

### Cell isolation and characterization

Adipose-derived stem cells (ADSCs) were extracted from epididymal adipose tissue of 2-week-old Sprague–Dawley (SD) rats as previously described [13]. Cells were centrifuged at 1500g for 5 min to remove impurities and then cultured in low-glucose DMEM medium with 10% fetal bovine serum in an incubator with 5% CO_2_ and 37℃. The fourth and fifth passages of ADSCs were selected for the experiment. CD29, CD90, CD34, CD11b, CD45 and CD14 antibodies were used to identify the surface markers of ADSCs by flow cytometry. Special stem cell differentiation medium was used to induce ADSCs to differentiate into adipocytes or osteoblasts. After 2 weeks, oil red O staining solution was used to identify adipocytes while alizarin red S staining was used to identify osteoblasts. The in *vitro* experiments were divided into three groups: ADSCs cultured in low glucose DMEM, supplemented with 10% FBS, 1% penicillin, and 1% Streptomycin (ADSCs group), and the other two groups were supplemented with 10μM Nrf2 activator tBHQ (tBHQ group) or 10μM Nrf2 inhibitor ML385 (ML385 group) in addition to the previous medium.

### Measurement of cell death and viability

The three groups of ADSCs were cultured in *vitro* for three days, then the medium was replaced with low-glucose DMEM containing 200μM hydrogen peroxide for two hours. The well plates were washed with PBS solution, and 1ml Calcein AM/PI detection solution was added. After incubated for half hour at 37℃ in a humidified atmosphere containing 5% CO_2_, the proportions of cells alive or dead were detected by fluorescent enzyme labeling (Calcein AM is green fluorescence, Ex/Em = 495/517nm; PI is red fluorescence, Ex/Em = 535/617nm).

### Real-time PCR

TRIzol kit (lnvitrogen) was used to extract RNA from the three groups of ADSCs. PrimeScript® RT reagent Kit (TaKaRa) was used to reverse transcription of RNA. The ABI PRISM 7000 sequence detector (Applied Biosystems) was used to perform Real-time PCR according to the instruction of SYBR® Premix Ex Taq™ (Perfect Real Time) (TaKaRa). Factors associated with oxidative stress, containing Nrf2 and superoxide Dismutase 1 (SOD1) were detected. Glyceraldehyde-phosphate dehydrogenase (GAPDH) gene was used as internal control. The primers used for analysis were shown in Table 1.

**Table 1:** Primer sequences for real-time PCR used in the study

Gene	Forward/Reverse	Sequence
Nrf2	F	CTGTCAGCTACTCCCAGGTTG
	R	TGGGAATATCCAGGGCAAGC
SOD1	F	TGGGGACAATACACAAGGCTG
	R	ATGCCTCTCTTCATCCGCTG
GAPDH	F	GGCATGGACTGTGGTCATGA
	R	TGATGGGTGTGAACCACGAG

### Cell labeling and transplantation

The three groups of ADSCs were mixed with red dye tracer PKH26 (1 × 10^6^ cells per 100μl suspension, Catalogue. # Mini 26, Sigma Chemical Co.) according to the manufacturer’s protocol, as previous described [14]. The PKH-26 labeled cells were transplanted around the major pelvic ganglion (MPG) of rats and detected on Day 3 and 7, respectively.

### Animals treatments

**Fig. 1:**
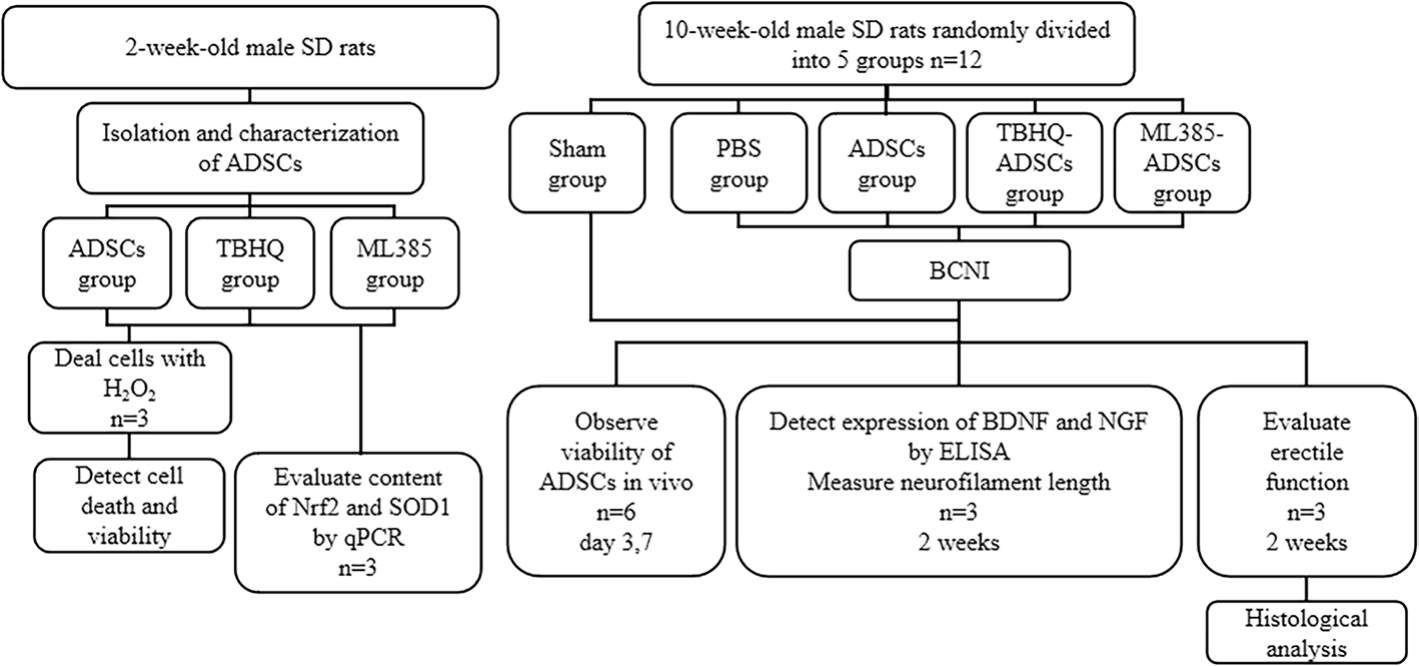
Flowchart of this study. ADSCs: Adipose-derived stem cells; BCNI: Bilateral cavernous nerve injury; BDNF: Brain derived neurotrophic factor; H_2_O_2_: hydrogen peroxide; Nrf2: Nuclear factor-E2-related Factor; NGF: Nerve growth factor; SD: Sprague–Dawley; SOD1: Superoxide Dismutase 1; TBHQ: Tert-Butylhydroquinone

Both two-week-old and ten-week-old male SD rats were obtained from the Experimental Animal Center of Sun Yat-sen University. As presented in Fig. 1, the two-week-old rats were used for extraction and isolation of ADSCs. Totally 60 ten-week-old SD rats were randomly divided into five groups (*n* = 12 rats per group). Among them, the Sham group received sham surgery, while the other four groups received bilateral cavernous nerve injury (BCNI) surgery and then were injected at the site of major pelvic ganglion (MPG) with the following respectively: PBS (PBS group), ADSCs (ADSCs group), ADSCs dealt with tBHQ (tBHQ-ADSCs group) or ADSCs dealt with ML385 (ML385-ADSCs group). To build the BCNI rat model, rats were anaesthetized with 2.5%-3% isoflurane. After skin preparation, the abdominal cavity was opened layer by layer and the bladder was exposed, then the MPG was found on the dorsal side of the prostate. The bilateral cavernous nerves (CN) were crushed at 1 mm distal to MPG for 2 min with a non-serrated hemostat [15]. After establishment of BCNI model, 100μl PBS containing 1 × 10^6^ PKH-26 labeled cells were injected around bilateral MPG. As a contrast, rats in the sham group received open surgery, but the CNs were not damaged, and 100μl PBS was injected around bilateral MPG.

### Enzyme-linked immunosorbent assay (ELISA)

Two weeks after BCNI surgery, three SD rats in each group were selected randomly, and then the MPG was extracted and rinsed 3 times for 1 min with PBS containing 1% penicillin–streptomycin. The rinsed MPG was placed in a 24-well plate for 5 min, and the 200μl MPG medium (BD substrate + RPMI1640 medium was mixed in a ratio of 1:2) was added. After 3 days of culture in a 37℃ incubator, the supernatants were collected from each group and detected using an ELISA kit (Enzyme Immunoassay Company) according to the manufacturer’s protocol. Briefly, the standard curve function was determined and calculated after adding samples. The absorbance value of the sample was determined and the brain derived neurotrophic factor (BDNF) and nerve growth factor (NGF) concentrations were calculated.

### Measurement of neurofilament growth of MPG

As described above, after extraction and culture for 3 days, the situation of MPG colonization and peripheral neurofilament growth were observed under microscope. The length of the nerve filament was measured to evaluate the neurofilament growth of MPG, by determining the distance between two points: the initial site where it originated from the MPG and its ultimate endpoint.

### Erectile function evaluation

Two weeks after BCNI surgery, the SD rats were anesthetized again with 2.5% isoflurane. The right carotid artery was exposed through a median incision from the chest to the neck. A heparinized PE-50 catheter was inserted to measure the mean arterial pressure (MAP). The MPG and CN were exposed again through a median lower abdominal incision. A heparinised 23-gauge needle was inserted into the penile crus after exposing the penis. The needle was connected to the BL-420 biological function system (Chengdu Taimeng Technology Ltd), and the CN was stimulated to record the Intracavernous pressure (ICP). The electrical stimulation parameters were 1.5 mA, 20 Hz, pulse width 0.2ms, and 50s duration. During tumescence, the maximal ICP (mICP) and total ICP (tICP, area under the curve) were recorded. The ratios of mICP and tICP to MAP were calculated to evaluate erectile function.

### Histological examination

Two weeks after BCNI surgery, the MPG and penis tissues of rats in each group were harvested and fixed with 4% paraformaldehyde. The tissues were prepared as 10μm frozen slices for immunofluorescence assay. The MPG tissue sections were incubated with primary antibody against S100β (Abcam; 1:200). The secondary antibody was Goat Anti-Mouse lgG(H + L) CY3 (Affinity Biosciences; 1:200), and the nuclei were stained with DAPI (Servicebio). Images were visualized and acquired using a confocal laser scanning microscope.

The ratio of smooth muscle (SM) to collagen in the corpus cavernosum was assessed by Masson's trichrome staining as previously described [16]. Images were quantified using Image J k 1.45 (National Institutes of Health).

### Statistical analysis

GraphPad Prism (version 9, GraphPad Software) was used for statistical analysis. Data were reported as means ± standard deviations (SD). If the parameters followed a normal distribution and exhibit homoscedasticity, a two-sample independent t-test was employed for comparing two groups, while analysis of variance (ANOVA) was used for comparing multiple groups. In cases where normal distribution and homoscedasticity assumptions were not met, the Wilcoxon rank-sum test or Kruskal–Wallis test was used for hypothesis testing.

## Results

### Isolation and Characterization of ADSCs

**Fig. 2:**
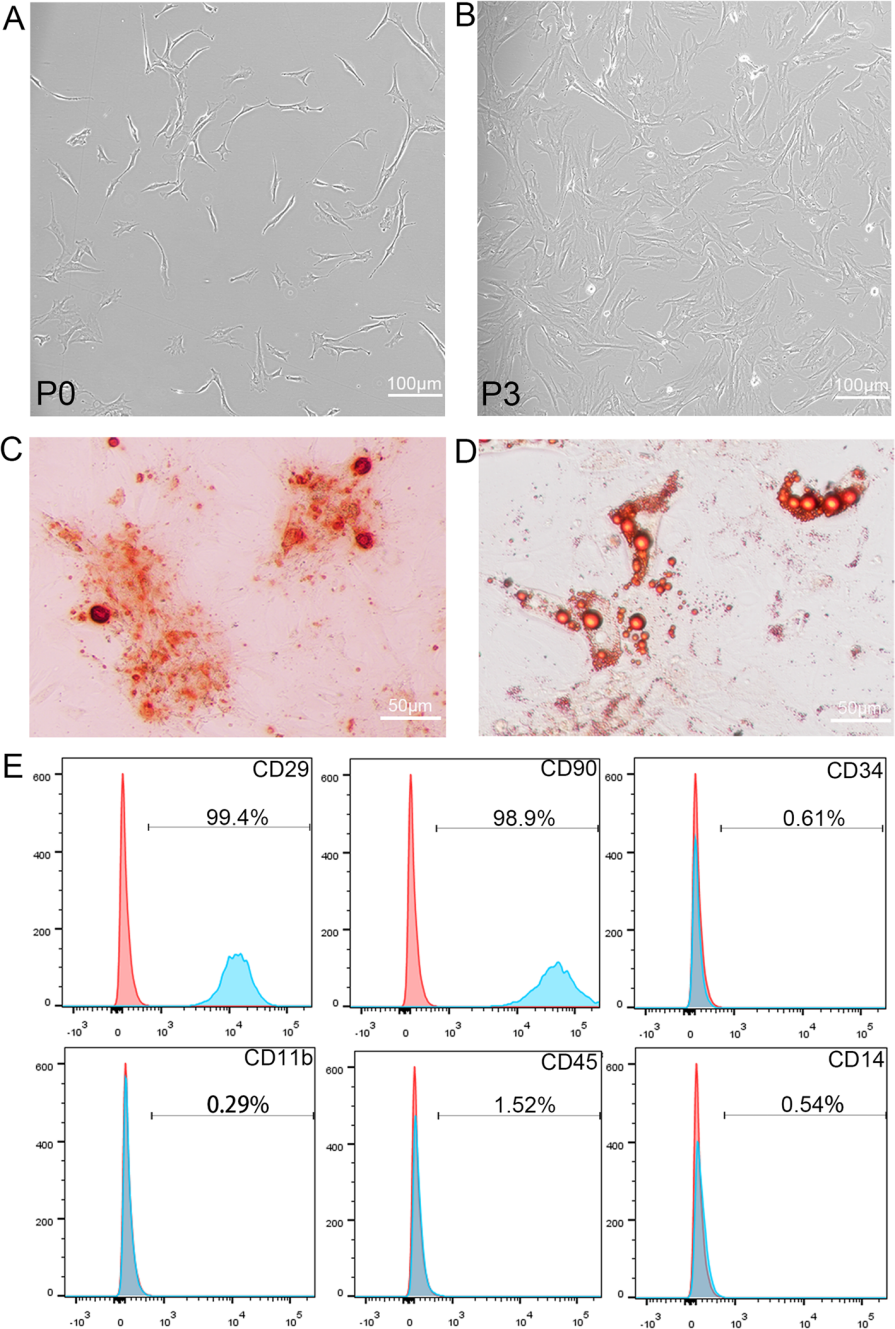
Isolation and Characterization of ADSCs. **A**,**B** Morphology of ADSCs in Passage 0 and Passage 3. **C**,**D** After induction, ADSCs presented typical phenotype of osteocytes (stained with Alizarin Red S) and adipocytes (stained with Oil Red O). **E** Flow cytometry showed that the ADSCs expressed stem cell markers (CD29, CD90), but not haematopoietic or endothelial markers (CD34, CD11b, CD45, CD14)

ADSCs presented a spindle or fibroblast-like morphology (Fig. 2A, B). The osteogenic and adipogenic differentiation capacity was evaluated to determine the pluripotency of ADSCs. The results showed that cells were positive for alizarin red staining 2 weeks after induction, and positive for oil red O staining 4 weeks after induction (Fig. 2 C, D). In addition, the results of flow cytometry showed that the cells highly expressed known stem cell markers: CD29(99.4%), CD90(98.9%), but not haematopoietic or endothelial markers: CD34(0.61%), CD11b (0.29%), CD45(1.52%), CD14(0.54%) (Fig. 2E).

### TBHQ and ML385 affected the expression of Nrf2 and SOD1

**Fig. 3:**
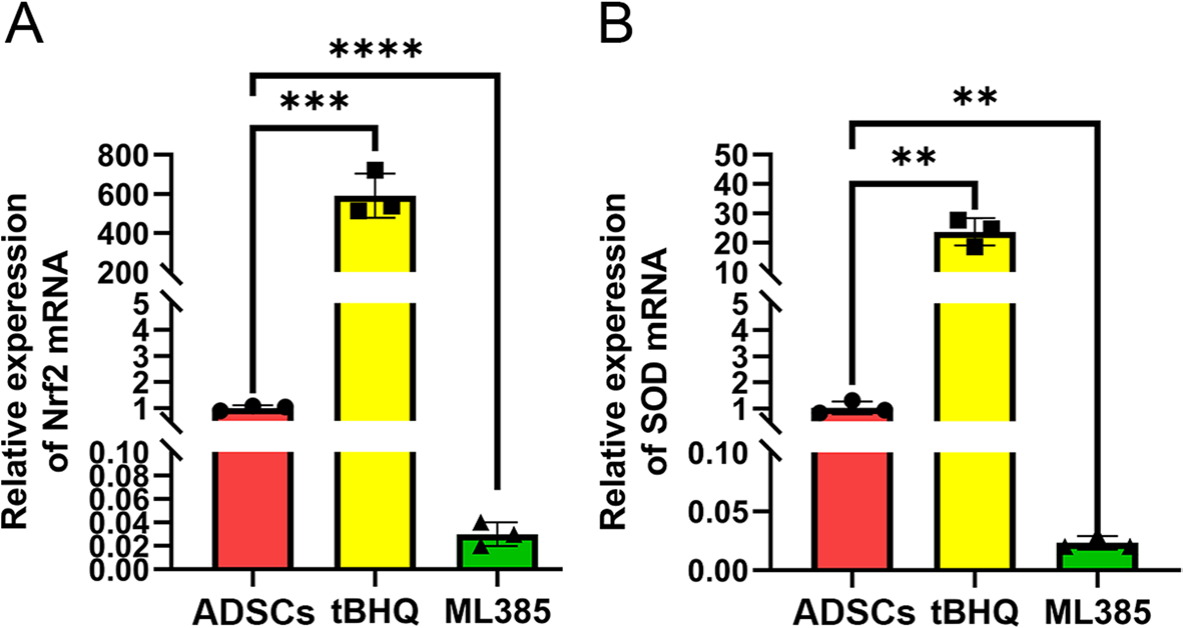
TBHQ and ML385 affected the expression of Nrf2 and SOD1. Real-time PCR revealed that compared with the ADSCs group, the expression level of Nrf2 and SOD1in tBHQ-ADSCs group increased significantly, while the expression level of Nrf2 and SOD1 in the ML385-ADSCs group decreased obviously. Each bar depicts the mean ± SD (*n* = 3). (****P < 0.0001, ***P < 0.001, **P < 0.01 and *P < 0.05)

Real-time PCR was performed to reveal the express level of factors associated with oxidative stress. As shown in Fig. 3, the expression levels of Nrf2 and SOD1 in the tBHQ group were significantly higher than those in both ADSCs and ML385 groups (*P* < 0.05). In contrast, the expression levels of Nrf2 and SOD1 in the ML385 group were significantly lower than those in the other two groups (*P* < 0.05).

### Nrf2 enhanced the ability of ADSCs to resist oxidative stress

**Fig. 4:**
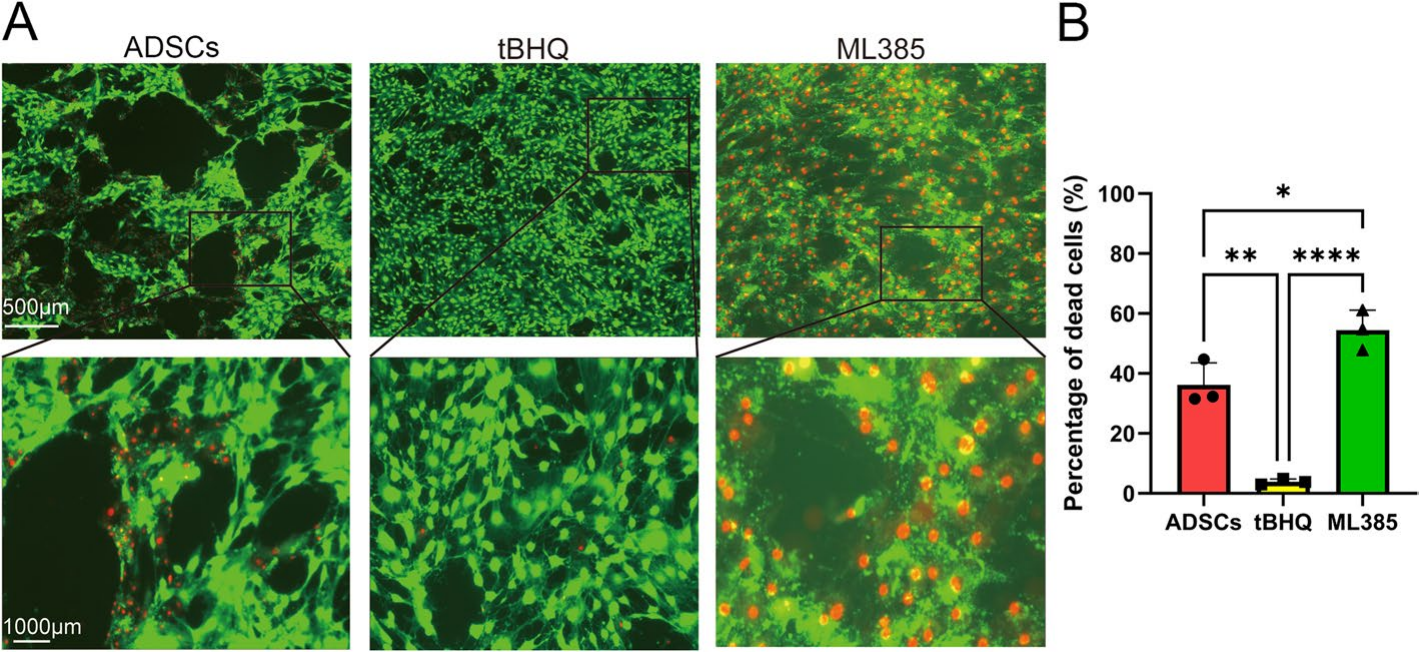
Nrf2 enhanced the ability of ADSCs to resist oxidative stress. **A** ADSCs in different groups were examined by Cell Viability/ Cytotoxicity Detection. The results showed alive cells in green and dead cells in red after H_2_O_2_ intervention. **B** Quantitative analysis of dead cells in different groups. The results revealed that the percentage of dead cells in the tBHQ group was significantly lower than those of ADSCs and ML385 groups. Each bar depicts the mean ± SD(*n* = 3). (****P < 0.0001, ***P < 0.001, **P < 0.01 and *P < 0.05)

After treated with hydrogen peroxide, ADSCs in different groups were examined by Cell Viability/ Cytotoxicity Detection. As shown in Fig. 4, in the ADSCs group, the average number of alive cells and dead cells was 1745 and 1005, respectively. While in the tBHQ group, the number of alive and dead cells was 713 and 32, with a significant decrease in dead cell proportion (*P* < 0.05). On the contrary, in the ML385 group, the data was 2046 and 2406, with a great increase in dead cell proportion (*P* < 0.05).

### Nrf2 increased survival ability of ADSCs in vivo

**Fig. 5:**
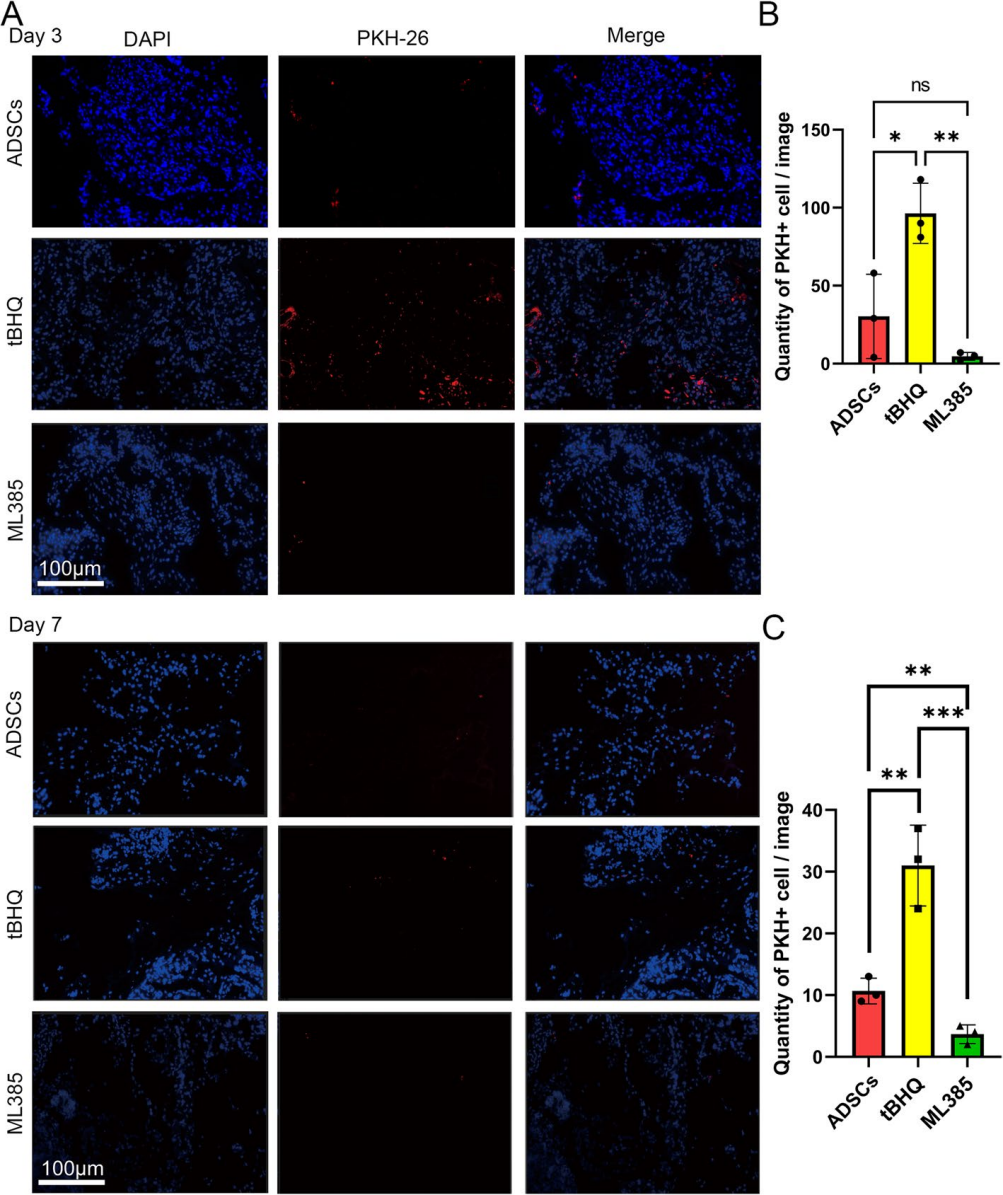
Nrf2 increased survival ability of ADSCs in vivo. **A** PKH26-labled cells were detectable on Day 3 and Day 7 after implantation in all three groups. **B**,**C** The quantity of PKH-26 + cells on Day 3 (B) and Day 7 (C) in different groups were analyzed by Image J. The results revealed that the tBHQ group presented higher quantity of PKH-26 + cells compared to both ADSCs and ML385 groups. Each bar depicts the mean ± SD (*n* = 3). (****P < 0.0001, ***P < 0.001, **P < 0.01 and *P < 0.05)

The PKH-26-labeled cells were injected around the MPG in vivo and the immunofluorescence technique was used to observe the survival of cells both 3 and 7 days after transplantation. As shown in Fig. 5, PKH26 + cells were detectable in all three groups either on Day 3 or 7 after transplantation. The quantity of PKH26 + cell in the tBHQ group was higher than the other groups on both Day 3 and 7 (*P* < 0.05).

### Nrf2 improved efficiency of ADSCs in nerve component restoration and secretion ability of MPG

**Fig. 6:**
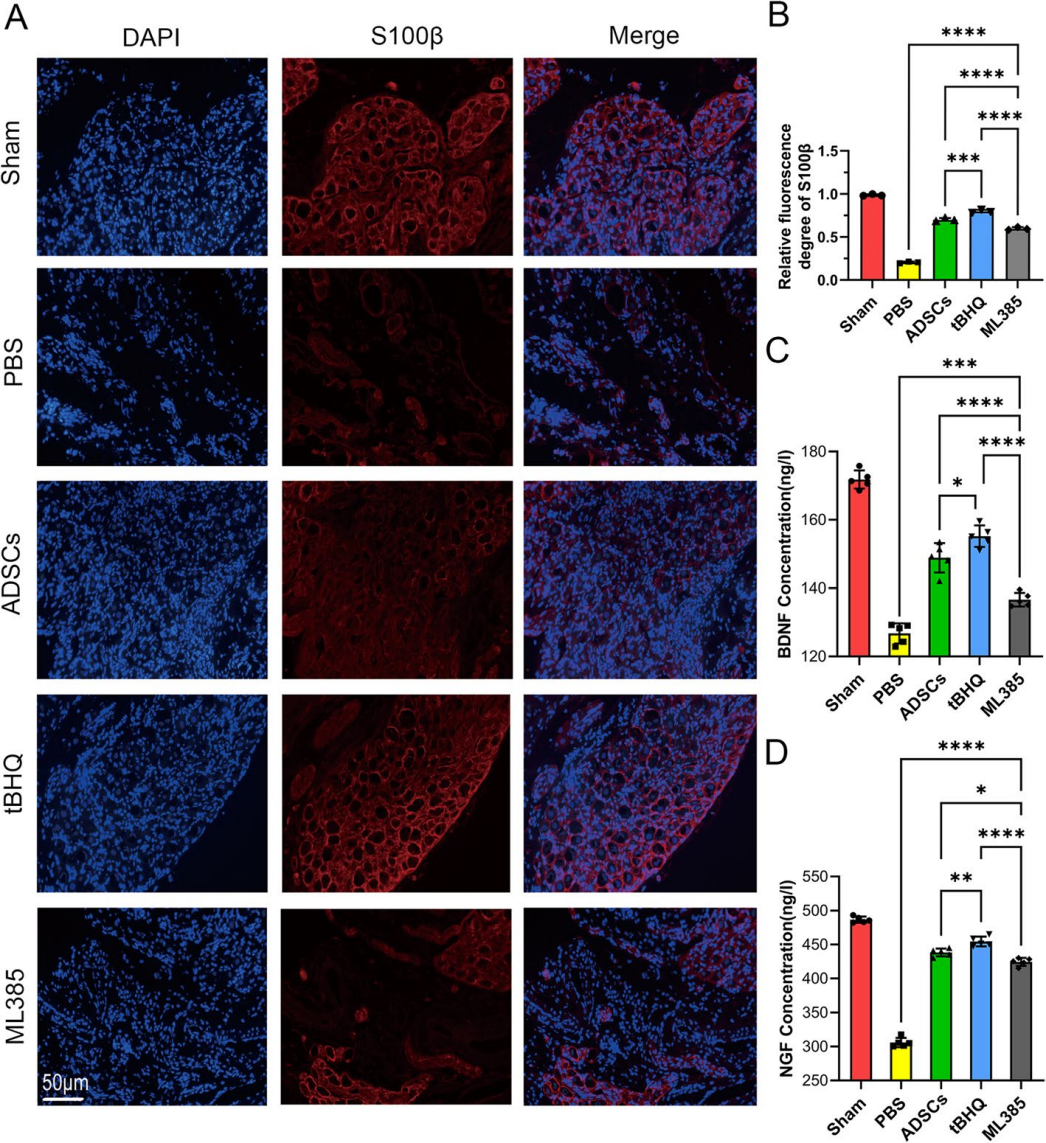
Nrf2 improved efficiency of ADSCs in nerve component restoration and secretion ability of MPG. **A** S100β (red) immunofluorescence staining of MPG tissues from rats in each experimental group. **B** Quantitative analysis of immunofluorescence results for S100β. The results revealed that the expression of S100β decreased obviously in the PBS group, and it was ameliorated most obviously in the tBHQ-ADSCs group. **C**,**D** Quantitative analysis of BDNF and NGF concentrations in the MPG culture supernatant in each experimental group. Compared with the PBS group, the expressions of BDNF and NGF in the ADSCs, tBHQ and ML385-ADSCs groups increased significantly, especially in the tBHQ-ADSCs group. Each bar depicts the mean ± SD(*n* = 3). (****P < 0.0001, ***P < 0.001, **P < 0.01 and *P < 0.05)

Immunofluorescence staining was used to investigate the expression of S100β in the MPG 2 weeks after cells transplantation. The results showed that the expression of S100β decreased obviously after BCNI surgery in the PBS group, and it was ameliorated most obviously in the tBHQ-ADSCs group. Moreover, the expression of S100β in the ML385-ADSC group were lower than those in both ADSCs and tBHQ-ADSCs groups(*P* < 0.05) (Fig. 6A, B).

**Fig. 7:**
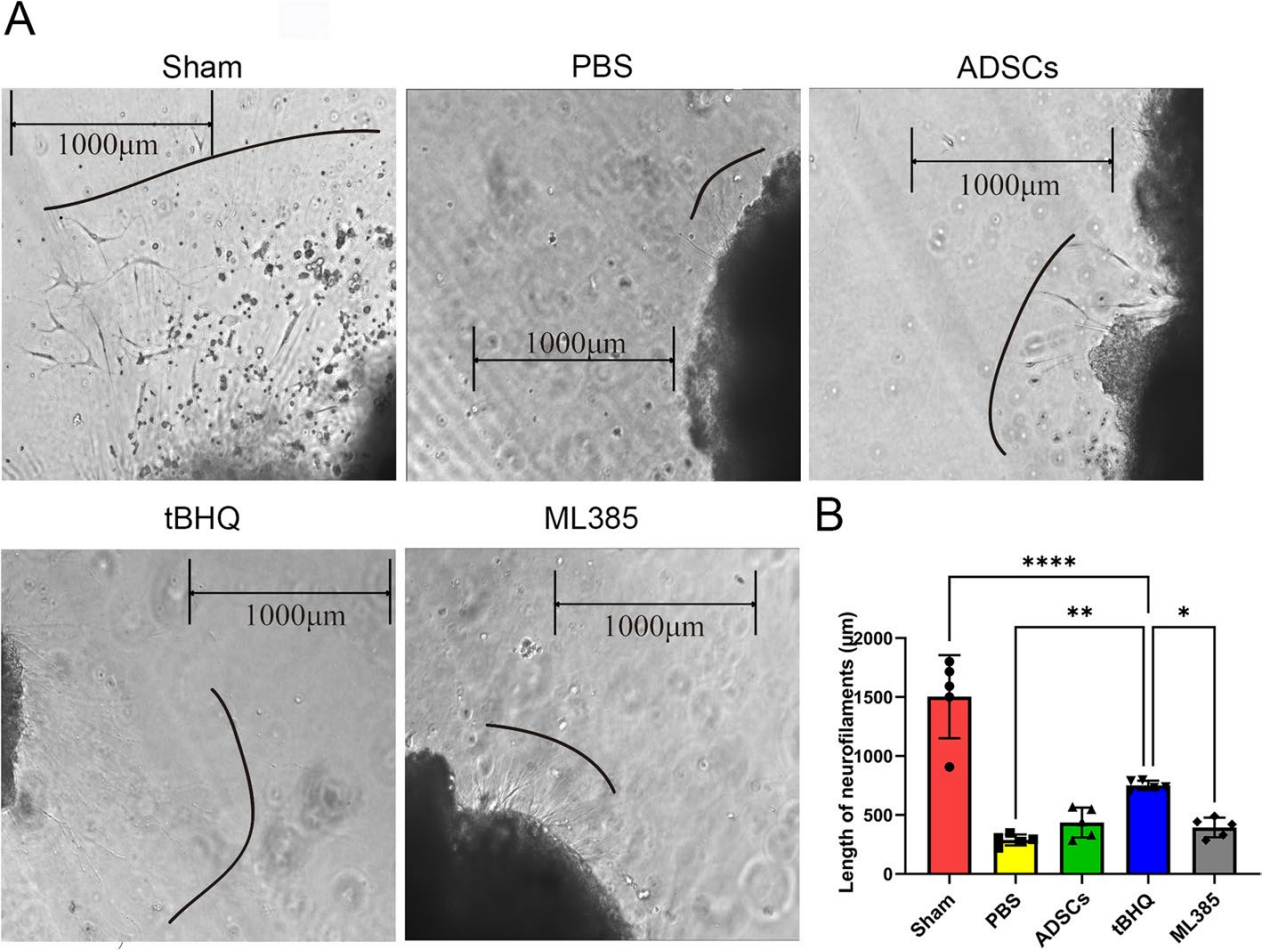
Nrf2 improved efficiency of ADSCs in MPG nerve regeneration. A: The neurofilament growth of the MPG in each experimental group. (B) Quantitative analysis of the neurofilament growth length of the MPG in each experimental group. The length was measured from the initial site and its ultimate endpoint (the endpoint was marked as a black line). The result revealed that the neurofilament length was longer in the tBHQ-ADSCs group than those of the ML385-ADSCs and PBS groups. Each bar depicts the mean ± SD (*n* = 4). (****P < 0.0001, ***P < 0.001, **P < 0.01 and *P < 0.05)

Two weeks after cell transplantation, MPG of each group was extracted and cultured in vitro for 3 days. ELISA kit was used to detect the effect of transplanted ADSCs on the secretion of neurotrophic factors by MPG. The data showed that compared with the sham group, the expressions of BDNF and NGF decreased after BCNI surgery. Compared with the PBS group, treatment with different ADSCs elevated expression of BDNF and NGF, especially in the tBHQ-ADSCs group (*P* < 0.05). In addition, the expression of BDNF and NGF in the ML385-ADSCs group were lower than those in both ADSCs and tBHQ-ADSC groups (*P* < 0.05) (Fig. 6C, D). The growth length of neurofilament in MPG was detected under microscope. The results revealed that the growth length of neurofilament in the tBHQ-ADSCs group (751.32 ± 40.21 µm) was longer than those in both ML385-ADSC (393.90 ± 85.16 µm) and PBS group (288.55 ± 46.21µm) (*P* < 0.05), while there was no significant difference with the ADSCs group (435.51 ± 126.53) (Fig. 7).

### Nrf2 enhanced ability of ADSCs in restoration of erectile function in BCNI rats

**Fig. 8:**
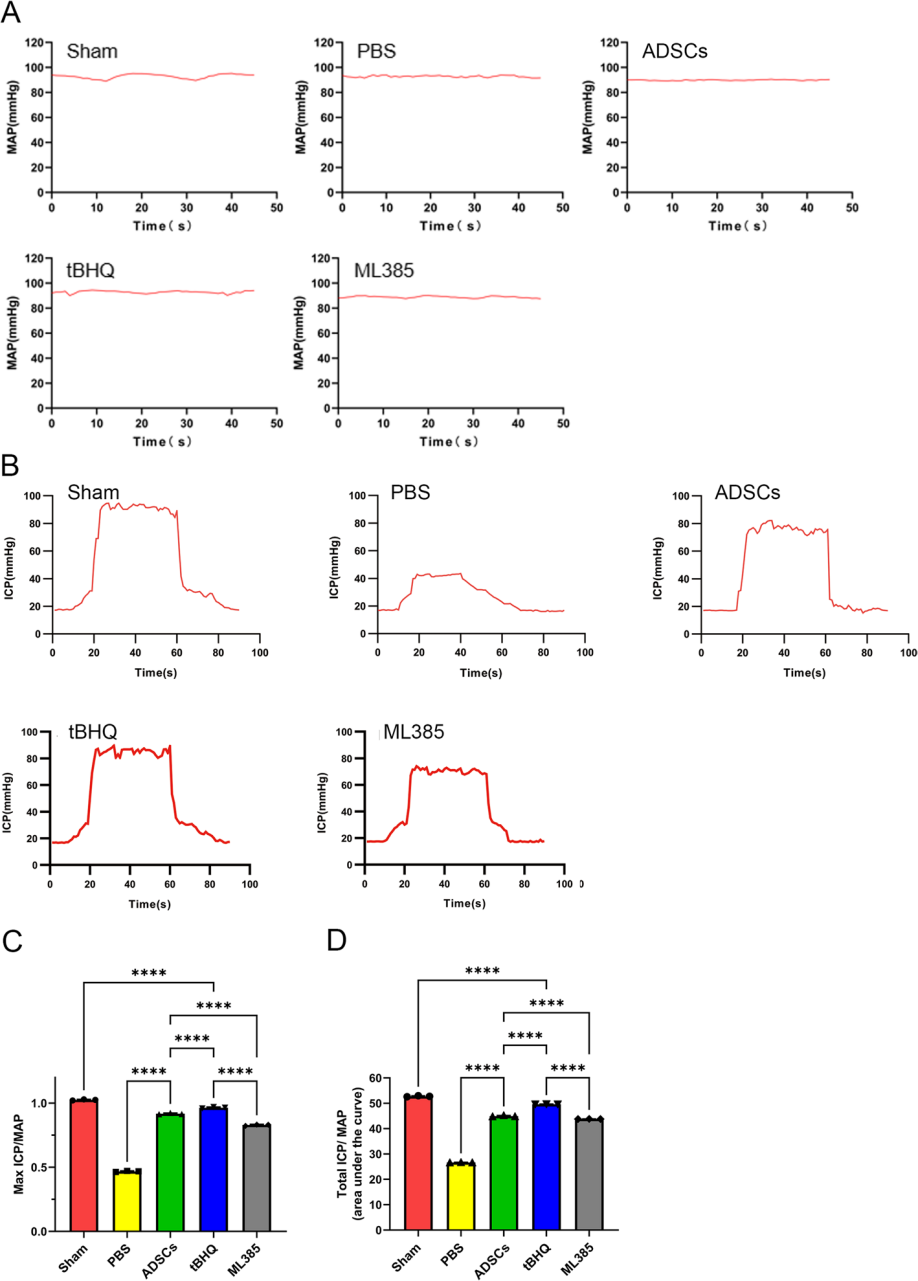
Nrf2 enhanced ability of ADSCs in restoration of erectile function in BCNI rats. **A** Standard MAP in each experimental group. **B** ICP responded to electrostimulation in each experimental group. **C**,**D** Maximum and total ICP to MAP ratios in the five groups.. Compared with the sham group, the PBS group revealed a significant decrease in both mICP/MAP and tICP/MAP ratios. The tBHQ-ADSCs group exhibited higher mICP/MAP and tICP/MAP ratios compared to the ADSCs and ML385-ADSCs groups. Each bar depicts the mean ± SD (*n* = 3). (****P < 0.0001, ***P < 0.001, **P < 0.01 and *P < 0.05). ICP: Intracavernous pressure; MAP: Mean arterial pressure

Erectile function was evaluated by electrical stimulation of the CN two weeks after CNI and cell transplantation. There were no significant differences in MAP among the five groups (Fig. 8A). The typical ICP curve of each group was presented in Fig. 8B. Compared with the sham group (52.74 ± 0.01 and 90.79% ± 0.20%), the PBS group revealed a significant decrease in both mICP/MAP and tICP/MAP ratios (26.67 ± 0.00 and 39.53% ± 0.29%, *P* < 0.05). The mICP/MAP and tICP/MAP ratios increased after cell transplantation in the three groups. In detail, the tBHQ-ADSCs group (49.65 ± 0.00 and 85.40% ± 0.07%) exhibited higher mICP/MAP and tICP/MAP ratios compared to the ADSCs (44.93 ± 0.00 and 79.32% ± 0.32%) and ML385-ADSCs (43.80 ± 0.00 and 73.14% ± 0.05%) groups (Fig. 8C, D).

### Nrf2 enhanced ability of ADSCs in prevention of fibrosis of the corpus cavernosum

**Fig. 9:**
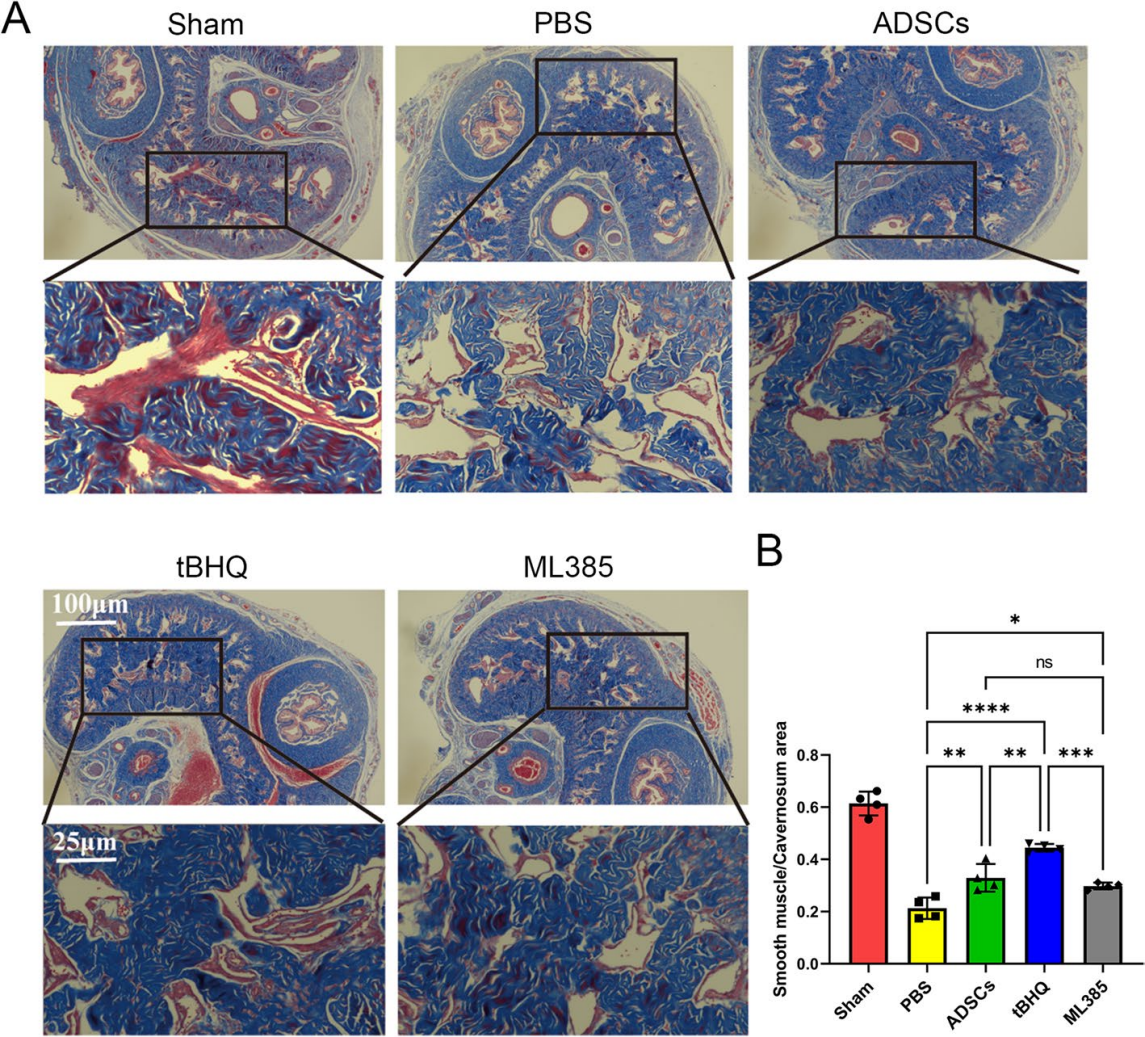
Nrf2 enhanced ability of ADSCs in preventing fibrosis in the corpus cavernosum and increasing cavernosal smooth muscle content. **A** Representative images of Masson trichrome staining in penile specimen. Smooth muscle and collagen were stained in red and blue, respectively. **B** Quantitative analysis of smooth muscle and collagen ratios in each group. The ratio decreased significantly in the PBS group. In addition, the tBQH-ADSCs group exhibited higher ratio than both ADSCs and ML385-ADSCs groups. Each bar depicts the mean ± SD (*n* = 4). (****P < 0.0001, ***P < 0.001, **P < 0.01 and *P < 0.05)

Masson staining was used to measure the ratio of SM to collagen composition in the corpus cavernosum. Transplantations of ADSCs restored the ratio of (SM) to collagen in CNI rats. As shown in Fig. 9, after BCNI surgery, the SM/collagen ratio decreased significantly in the PBS group (21.28% ± 4.10%). In addition, the tBQH-ADSCs group (44.48% ± 1.45%) exhibited higher SM/collagen ratio than both ADSCs (32.92% ± 5.30%) and ML385-ADSCs group (29.78% ± 1.31%), while there was no significant difference between the ML385-ADSCs and ADSCs group.

## Discussions

The occurrence of erection depends on the coordination of the nervous, vascular, and muscular systems [17]. During pelvic surgery, the pelvic autonomic nerve is prone to damage due to traction, compression or thermal injury, leading to ED [18]. PDE5I is currently the most commonly used drug in the clinical treatment of ED. These drugs primarily exert their therapeutic effects by dilating and relaxing the penile blood vessels, making them effective against ED caused by insufficient penile blood flow. However, the efficacy of treating nerve injury related ED after pelvic surgery is limited, which is primarily neurogenic. Consequently, many experimental studies are currently exploring potential alternative treatments. Among these approaches, stem cell therapy stands out as one of the most promising and effective methods. Previous studies applied different stem cells, containing ADSCs to treat (BCNI) rat models, proving that they can effectively alleviate ED [19]. However, some clinical trials have shown that stem cell therapy has not consistently achieved sustained long-term therapeutic effects in the treatment of ED. Recent studies also proved that stem cell therapy could improve erectile function early on, but in the later stages, erectile function tended to worsen [20–24]. Additionally, there was research indicating that while stem cell therapy offered some degree of improvement in penile rigidity, patients still could not complete vaginal penetration [25]. These studies suggest that further improvements in ED therapy with stem cells are in urgent need.

One important reason for the unsatisfactory therapeutic effect of MSCs is the low cell activity in vivo. It has been proved that oxidative stress injury (OSI) can significantly reduce the activity of MSCs [26, 27]. The survival rate of MSCs was easily reduced by ROS produced during in *vitro* culture. Moreover, hypoxia-induced ROS in the microenvironment of the injury site also largely affected the cell viability of MSCs after transplantation. Therefore, improving the anti-OSI ability of MSCs should be conductive to improve their activity and enhance their ability to repair nerve injury and ED.

Nrf2 is a key transcription factor that regulates cellular anti-oxidative stress. Under stress, Nrf2 is released into the nucleus through the Keap1-Nrf2 pathway and plays a role in anti-oxidative stress [28]. Therefore, in this study, tBHQ and ML385 were used to regulate the activity of Nrf2 in ADSCs and alter the anti-oxidative stress ability of ADSCs [29–32]. Both functional and morphological observations showed that the tBHQ group promoted CN repair, thereby improving erectile function and reducing penile fibrosis. However, the effect of CN repair was decreased in the ML385 group, and the improvement of erectile function was weakened. Furthermore, the Real-time PCR results showed that the expression levels of Nrf2 and SOD1 were increased in the tBHQ group. Mechanistically, Nrf2 regulation alters the ability of ADSCs to resist oxidative stress.

The pelvic ganglion gives off branches of the CN, and the function of the pelvic ganglion is also affected when the CN is crushed [33]. Therefore, in the BCNI model, the secretion capacity and content of neurotrophic factors in the pelvic ganglia, as well as the regeneration ability are important indicators to evaluate the therapeutic effect of ADSCs injection on CN. BDNF and NGF are important neurotrophins that play an important role in synaptic development and plasticity [34–36]. The secretion levels of BDNF and NGF in the tBHQ-ADSCs group were higher than those in the ADSCs group, while those in ML385-ADSCs group were lower than those in the ADSCs group. S100β, a neuronal myelin marker, indicates peripheral nerve myelination [37]. The expression trend of S100β in pelvic ganglia was the same in the tBHQ-ADSCs and ML385-ADSCs groups. Therefore, we hypothesized that the nerve repair in the tBHQ-ADSCs group was stronger than in the ADSCs group, while neural repair in the ML385-ADSCs group was weaker than that in the ADSCs group. This proves that Nrf2 could positively regulate the neural repair ability of ADSCs.

The impairment of erectile function after nerve injury is partly due to changes in the smooth muscle and fibrous composition of the corpus cavernosum [38, 39]. The increase of fibrous composition leads to fibrosis of the corpus cavernosum, a process that is difficult to reverse. Therefore, the ratio of corpus cavernosum smooth muscle to corpus cavernosum fibers is an important evaluation criterion for the restoration of erectile function. We assessed erectile function and smooth muscle/fiber ratio two weeks after BCNI surgery. The fiber component in the PBS group was increased. Moreover, the smooth muscle/fiber ratio of the tBHQ-ADSCs group was higher than that of the ADSCs group, suggesting that the anti-fibrosis effect of the tBHQ-ADSCs group was better.

In this study, physiological detection was also applied to investigate the erectile function. As penile erection is closely related to the continuous increase and maintenance of intracavernous vascular pressure (ICP), the ratio of ICP to MAP can reflect the function of cavernous blood vessels during penile erection [40–43]. An increase in the total and maximal ICP/MAP in the tBHQ-ADSCs group suggested better erectile function than in the ADSCs group.

The current study has some limitations. First, the experimental results obtained from rat models might not be fully representative of the therapeutic effect in patients. Second, this study only explored the therapeutic effect of tBHQ-ADSCs and ML385-ADSCs on rat model for a period of 14 days. We may need extend different time points to observe the effect of CN repair in future research. Third, we used cell fluorescence density for statistical analysis in some cell experiments, which may be affected by cell proliferation and migration, and we would like to improve the detection methods in the future research. Last, since neurogenic ED is caused by loss of reception of neurotransmitters, such as dopaminergic and serotoninergic neurotransmitters, further studies should be performed to compare whether the ADSCs treatment has any relationship with effects on these receptors.

## Conclusions

In conclusion, our current study showed that Nrf2 could enhance anti-oxidative stress ability of ADSCs, and thus improved the therapeutic effect of ADSCs for neurogenic ED in the rat model. These findings will provide potential therapeutic methods for neurogenic ED and new insights for ADSCs in the mechanism of nerve-injured ED treatment.

## Data Availability

The datasets used and/or analyzed during the current study are available from the corresponding author on reasonable request.

## Abbreviations

ADSCs: Adipose-derived stem cells
BCNI: Bilateral cavernous nerve injury
BDNF: Brain derived neurotrophic factor
CN: Cavernous nerve
ED: Erectile dysfunction
ICP: Intracavernous pressure
MAP: Mean arterial pressure
MPG: Major pelvic ganglion
MSCs: Mesenchymal stem cells
NAD(P)H: Nicotinamide adenine dinucleotide phosphate
Nrf2: Nuclear factor-E2-related Factor
NGF: Nerve growth factor
OSI: Oxidative stress injury
PDE5Is: Phosphodiesterase type 5 inhibitors
SOD1: Superoxide Dismutase 1
SD: Sprague-Dawley
SM: Smooth muscle
TBHQ: Tert-Butylhydroquinone
